# Supplementary material for: Mutual information for detecting multi-class biomarkers when integrating multiple bulk or single-cell transcriptomic studies
Source: Bioinformatics. 2024 Nov 19;40(12):btae696. doi: 10.1093/bioinformatics/btae696 (PMC11629966; doi:10.1093/bioinformatics/btae696)
Supplement: btae696_Supplementary_Data [file btae696_supplementary_data.docx]

**SUPPLEMENTARY MATERIAL**

**Mutual information for detecting multi-class**

**biomarkers when integrating multiple bulk or**

**single-cell transcriptomic studies**

Contents:

1. **Fig. S1**: The boxplots for the averaged gene expression patterns of all the different gene categories across four tissues in the mouse metabolism study.
2. **Fig. S2**: The boxplot for the gene expression patterns of *Blvrb.*
3. **Fig. S3:** The boxplots for the averaged gene expression patterns of all the different gene categories across three leukemia studies.
4. **Table S1**: Simulation settings for different gene types.
5. **Table S2**: Simulation settings for different scenarios.
6. **Table S3**: Results of the simulation for different scenarios.
7. **Table S4**: Simulation for equal vs. unequally weighted settings
8. **Table S5**: IPA canonical pathway analysis on M1 and M4 genes in mouse metabolism data.
9. **Table S6**: LISA results for top 30 ranked transcription factors.
10. **Table S7**: Distribution of the number of cells for each cell type.
11. **Table S8:** List of immune-tumor discordant genes in TNBC tumor microenvironment.

**Fig. S1. The boxplots for the averaged gene expression patterns of all the different gene categories across four tissues in the mouse metabolism study.** V consists of genes detected by min-MCC only, while M1 represents the intersection of genes detected by both min-MCC and MICA. M2-M11 represent gene categories with concordance shared between different tissue pairs: M2 in brown fat, liver, and heart, M3 in brown fat, liver, and skeletal, M4 in brown fat, heart, and skeletal, and M5 in liver, heart, and skeletal, M6 in brown fat and liver, M7 in brown fat and heart, M8 in brown fat and skeletal, M9 in liver and heart, M10 in liver and skeletal, M11 in heart and skeletal.

**
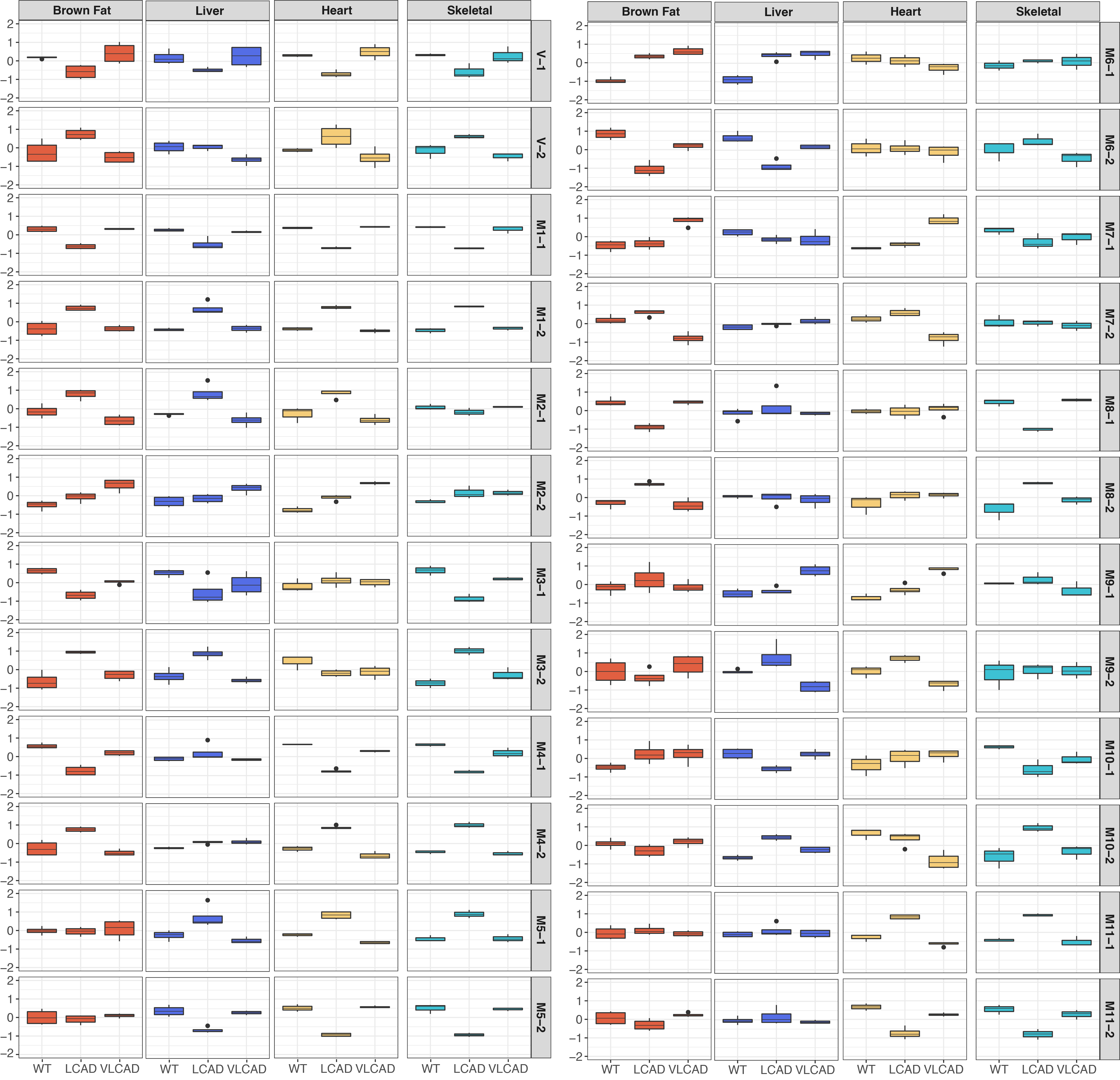
**

**Fig. S2. The boxplot for the gene expression patterns of *Blvrb*.** Concordance gene expression is in brown fat, heart and skeletal tissues.

**
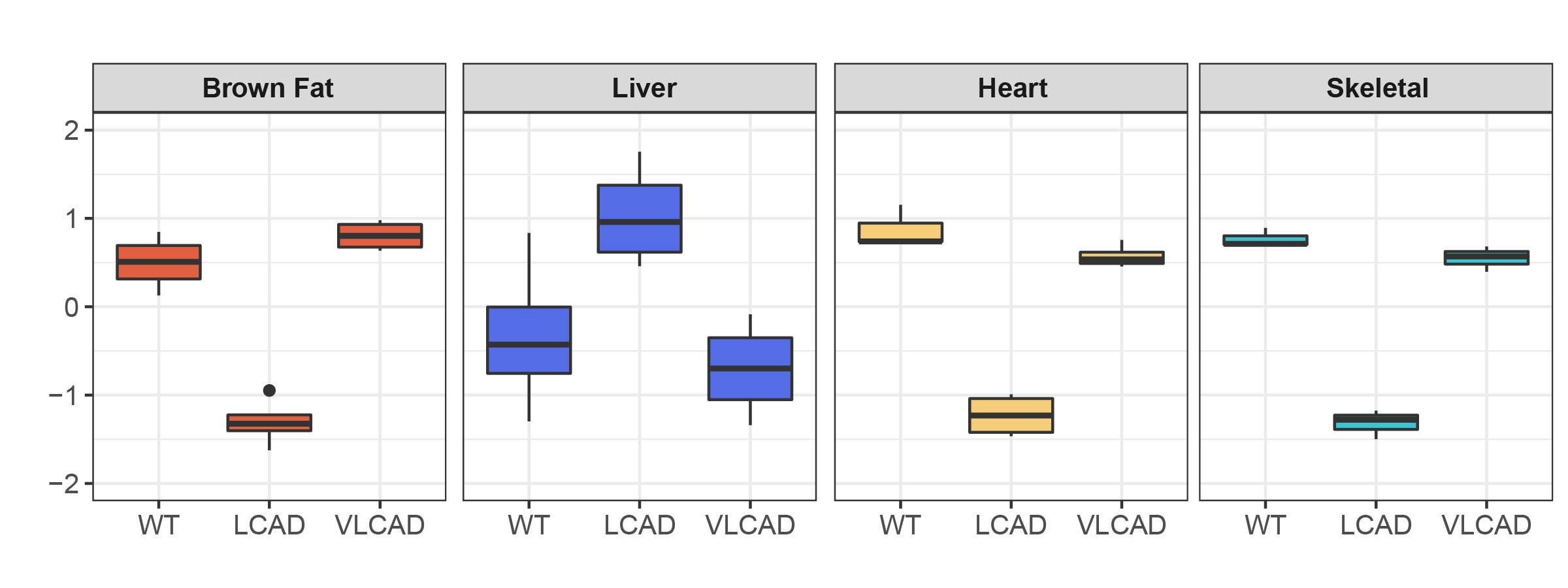
**

**Fig. S3. The boxplots for the averaged gene expression patterns of all the different gene categories across three leukemia studies.** The gene categories include the genes identified by min-MCC alone (V), the intersected genes identified by min-MCC and MSCA (M1), genes identified only by MSCA and the partial shared concordance detected in GSE6891 and GSE17855 (M2), concordance between GSE6891 and GSE13159 (M3), and concordance between GSE17855 and GSE13159 (M4).


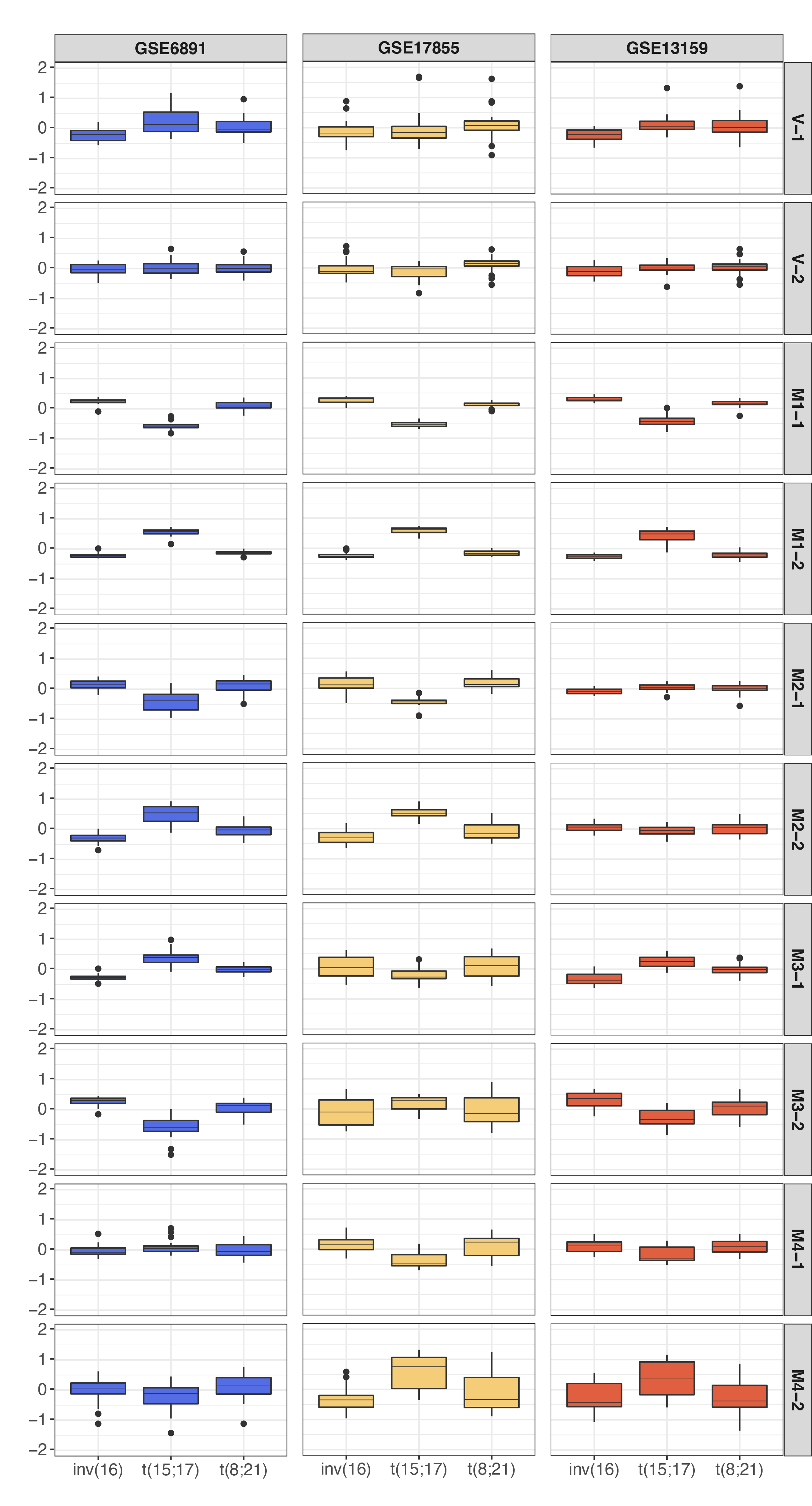


**Table S1**: **Simulation settings for different gene types.** Gene Type I: All four studies share the concordant signals; Gene Type II: concordant expression is seen in study 1, 2, and 3; Gene Type III: concordant expression is seen between study 1 and 2 and between 3 and 4; Gene Type IV: all four studies contain complete noises without any pattern. 500 replicates were simulated for each gene type.

|  | **Study 1** | **Study 2** | **Study 3** | **Study 4** |
| --- | --- | --- | --- | --- |
|  | (n_11_, n_12_, n_13_) =  (10,10,10) | (n_21_, n_22_, n_23_) =  (10,10,10) | (n_31_, n_32_, n_33_) =  (10,10,10) | (n_41_, n_42_, n_43_) =  (10,10,10) |
|  | (μ_11_, μ_12_, μ_13_), σ_1_ | (μ_21_, μ_22_, μ_23_), σ_2_ | (μ_31_, μ_32_, μ_33_), σ_3_ | (μ_41_, μ_42_, μ_43_), σ_4_ |
| Gene Type I | (1,3,5), 3 | (1,3,5), 3 | (1,3,5), 3 | (1,3,5), 3 |
| Gene Type II | (5,3,1), 3 | (5,3,1), 3 | (5,3,1), 3 | (1,7,1), 3 |
| Gene Type III | (1,3,5), 3 | (1,3,5), 3 | (1,7,1), 3 | (1,7,1), 3 |
| Gene Type IV | (0,0,0), 3 | (0,0,0), 3 | (0,0,0), 3 | (0,0,0), 3 |

**Table S2**: **Simulation settings for different scenarios.** Scenario 1: Sample size varies, with the number of samples within each class set to 5, 10, and 20. Scenario 2: Number of studies varies, set to 2, 4, and 8. Scenario 3: Number of classes varies, set to 2, 3, and 5. Scenario 4: Concordance levels vary, with $\rho=1, 0.8, 0.6, 0.5$.

| **Scenario 1:**  N = 5, 10, 20 | **Study 1** | **Study 2** | **Study 3** | **Study 4** |
| --- | --- | --- | --- | --- |
|  | (n_11_, n_12_, n_13_) =  (N, N, N) | (n_21_, n_22_, n_23_) =  (N, N, N) | (n_31_, n_32_, n_33_) =  (N, N, N) | (n_41_, n_42_, n_43_) =  (N, N, N) |
|  | (μ_11_, μ_12_, μ_13_), σ_1_ | (μ_21_, μ_22_, μ_23_), σ_2_ | (μ_31_, μ_32_, μ_33_), σ_3_ | (μ_41_, μ_42_, μ_43_), σ_4_ |
| Gene Type I | (1,3,5), 3 | (1,3,5), 3 | (1,3,5), 3 | (1,3,5), 3 |
| Gene Type II | (5,3,1), 3 | (5,3,1), 3 | (5,3,1), 3 | (1,7,1), 3 |
| Gene Type III | (1,3,5), 3 | (1,3,5), 3 | (1,7,1), 3 | (1,7,1), 3 |
| Gene Type IV | (0,0,0), 3 | (0,0,0), 3 | (0,0,0), 3 | (0,0,0), 3 |

| **Scenario 2:**  S = 2 | **Study 1** | **Study 2** |
| --- | --- | --- |
|  | (n_11_, n_12_, n_13_) =  (10,10,10) | (n_21_, n_22_, n_23_) =  (10,10,10) |
|  | (μ_11_, μ_12_, μ_13_), σ_1_ | (μ_21_, μ_22_, μ_23_), σ_2_ |
| Gene Type I | (1,3,5), 3 | (1,3,5), 3 |
| Gene Type II | (5,3,1), 3 | (5,3,1), 3 |
| Gene Type III | (1,3,5), 3 | (1,3,5), 3 |
| Gene Type IV | (0,0,0), 3 | (0,0,0), 3 |

| **Scenario 2:**  S = 4 | **Study 1** | **Study 2** | **Study 3** | **Study 4** |
| --- | --- | --- | --- | --- |
|  | (n_11_, n_12_, n_13_) =  (10,10,10) | (n_21_, n_22_, n_23_) =  (10,10,10) | (n_31_, n_32_, n_33_) =  (10,10,10) | (n_41_, n_42_, n_43_) =  (10,10,10) |
|  | (μ_11_, μ_12_, μ_13_), σ_1_ | (μ_21_, μ_22_, μ_23_), σ_2_ | (μ_31_, μ_32_, μ_33_), σ_3_ | (μ_41_, μ_42_, μ_43_), σ_4_ |
| Gene Type I | (1,3,5), 3 | (1,3,5), 3 | (1,3,5), 3 | (1,3,5), 3 |
| Gene Type II | (5,3,1), 3 | (5,3,1), 3 | (5,3,1), 3 | (1,7,1), 3 |
| Gene Type III | (1,3,5), 3 | (1,3,5), 3 | (1,7,1), 3 | (1,7,1), 3 |
| Gene Type IV | (0,0,0), 3 | (0,0,0), 3 | (0,0,0), 3 | (0,0,0), 3 |

| **Scenario 2:**  S = 8 | **Study 1** | **Study 2** | **Study 3** | **Study 4** |
| --- | --- | --- | --- | --- |
|  | (n_11_, n_12_, n_13_) =  (10,10,10) | (n_21_, n_22_, n_23_) =  (10,10,10) | (n_31_, n_32_, n_33_) =  (10,10,10) | (n_41_, n_42_, n_43_) =  (10,10,10) |
|  | (μ_11_, μ_12_, μ_13_), σ_1_ | (μ_21_, μ_22_, μ_23_), σ_2_ | (μ_31_, μ_32_, μ_33_), σ_3_ | (μ_41_, μ_42_, μ_43_), σ_4_ |
| Gene Type I | (1,3,5), 3 | (1,3,5), 3 | (1,3,5), 3 | (1,3,5), 3 |
| Gene Type II | (1,3,5), 3 | (1,3,5), 3 | (1,3,5), 3 | (1,3,5), 3 |
| Gene Type III | (1,3,5), 3 | (1,3,5), 3 | (1,3,5), 3 | (1,3,5), 3 |
| Gene Type IV | (0,0,0), 3 | (0,0,0), 3 | (0,0,0), 3 | (0,0,0), 3 |
|  | **Study 5** | **Study 6** | **Study 7** | **Study 8** |
|  | (n_11_, n_12_, n_13_) =  (10,10,10) | (n_21_, n_22_, n_23_) =  (10,10,10) | (n_31_, n_32_, n_33_) =  (10,10,10) | (n_41_, n_42_, n_43_) =  (10,10,10) |
|  | (μ_11_, μ_12_, μ_13_), σ_1_ | (μ_21_, μ_22_, μ_23_), σ_2_ | (μ_31_, μ_32_, μ_33_), σ_3_ | (μ_41_, μ_42_, μ_43_), σ_4_ |
| Gene Type I | (1,3,5), 3 | (1,3,5), 3 | (1,3,5), 3 | (1,3,5), 3 |
| Gene Type II | (1,3,5), 3 | (1,3,5), 3 | (1,3,5), 3 | (1,7,1), 3 |
| Gene Type III | (1,7,1), 3 | (1,7,1), 3 | (1,7,1), 3 | (1,7,1), 3 |
| Gene Type IV | (0,0,0), 3 | (0,0,0), 3 | (0,0,0), 3 | (0,0,0), 3 |

| **Scenario 3:**  K = 2 | **Study 1** | **Study 2** | **Study 3** | **Study 4** |
| --- | --- | --- | --- | --- |
|  | (n_11_, n_12_) =  (10,10) | (n_21_, n_22_) =  (10,10) | (n_31_, n_32_) =  (10,10) | (n_41_, n_42_) =  (10,10) |
|  | (μ_11_, μ_1_), σ_1_ | (μ_21_, μ_22_), σ_2_ | (μ_31_, μ_32_), σ_3_ | (μ_41_, μ_42_), σ_4_ |
| Gene Type I | (1,3), 3 | (1,3), 3 | (1,3), 3 | (1,3), 3 |
| Gene Type II | (5,3), 3 | (5,3), 3 | (5,3), 3 | (1,7), 3 |
| Gene Type III | (1,3), 3 | (1,3), 3 | (1,7), 3 | (1,7), 3 |
| Gene Type IV | (0,0), 3 | (0,0), 3 | (0,0), 3 | (0,0), 3 |

| **Scenario 3:**  K = 3 | **Study 1** | **Study 2** | **Study 3** | **Study 4** |
| --- | --- | --- | --- | --- |
|  | (n_11_, n_12_, n_13_) =  (10,10,10) | (n_21_, n_22_, n_23_) =  (10,10,10) | (n_31_, n_32_, n_33_) =  (10,10,10) | (n_41_, n_42_, n_43_) =  (10,10,10) |
|  | (μ_11_, μ_12_, μ_13_), σ_1_ | (μ_21_, μ_22_, μ_23_), σ_2_ | (μ_31_, μ_32_, μ_33_), σ_3_ | (μ_41_, μ_42_, μ_43_), σ_4_ |
| Gene Type I | (1,3,5), 3 | (1,3,5), 3 | (1,3,5), 3 | (1,3,5), 3 |
| Gene Type II | (5,3,1), 3 | (5,3,1), 3 | (5,3,1), 3 | (1,7,1), 3 |
| Gene Type III | (1,3,5), 3 | (1,3,5), 3 | (1,7,1), 3 | (1,7,1), 3 |
| Gene Type IV | (0,0,0), 3 | (0,0,0), 3 | (0,0,0), 3 | (0,0,0), 3 |

| **Scenario 3:**  K = 5 | **Study 1** | **Study 2** | **Study 3** | **Study 4** |
| --- | --- | --- | --- | --- |
|  | (n_11_, n_12_, n_13_, n_14_, n_15_) =  (10,10,10,10,10) | (n_21_, n_22_, n_23_, n_24_, n_25_) =  (10,10,10,10,10) | (n_31_, n_32_, n_33_, n_34_, n_35_) =  (10,10,10,10,10) | (n_41_, n_42_, n_43_, n_44_, n_45_) =  (10,10,10,10,10) |
|  | (μ_11_, μ_12_, μ_13,_ μ_14_, μ_15_), σ_1_ | (μ_21_, μ_22_, μ_23,_ μ_24_, μ_25_), σ_2_ | (μ_31_, μ_32_, μ_33,_ μ_34_, μ_35_), σ_3_ | (μ_41_, μ_42_, μ_43,_ μ_44_, μ_45_), σ_4_ |
| Gene Type I | (1,3,5,7,9), 3 | (1,3,5,7,9), 3 | (1,3,5,7,9), 3 | (1,3,5,7,9), 3 |
| Gene Type II | (9,7,5,3,1), 3 | (9,7,5,3,1), 3 | (9,7,5,3,1), 3 | (2,1,7,1,2), 3 |
| Gene Type III | (1,3,5,7,9), 3 | (1,3,5,7,9), 3 | (2,1,7,1,2), 3 | (2,1,7,1,2), 3 |
| Gene Type IV | (0,0,0,0,0), 3 | (0,0,0,0,0), 3 | (0,0,0,0,0), 3 | (0,0,0,0,0), 3 |

| **Scenario 4:**  $\rho=0.4, 0.6, 0.8, 1$ | **Study 1** | **Study 2** | **Study 3** | **Study 4** |
| --- | --- | --- | --- | --- |
|  | (n_11_, n_12_, n_13_, n_14_, n_15_) =  (10,10,10,10,10) | (n_21_, n_22_, n_23_, n_24_, n_25_) =  (10,10,10,10,10) | (n_31_, n_32_, n_33_, n_34_, n_35_) =  (10,10,10,10,10) | (n_41_, n_42_, n_43_, n_44_, n_45_) =  (10,10,10,10,10) |
|  | (μ_11_, μ_12_, μ_13,_ μ_14_, μ_15_), σ_1_ | (μ_21_, μ_22_, μ_23,_ μ_24_, μ_25_), σ_2_ | (μ_31_, μ_32_, μ_33,_ μ_34_, μ_35_), σ_3_ | (μ_41_, μ_42_, μ_43,_ μ_44_, μ_45_), σ_4_ |
| Gene Type I ($\rho=0.4$) | (3.32, 2.61, 1.63, 0.70, 1.73), 3 | (3.14, 1.56, 0.60, 1.96, 2.74), 3 | (2.01, 2.24, 1.60, 0.70, 3.45), 3 | (2.01, 3.37, 0.59, 1.76, 2.27), 3 |
| Gene Type II ($\rho=0.6$) | (1.72, 3.16, 2.60, 0.51, 2.00), 3 | (1.57, 3.35, 2.59, 1.76, 0.74), 3 | (2.73, 2.36, 3.04, 0.95, 0.92), 3 | (2.79, 3.33, 1.57, 1.1, 1.22), 3 |
| Gene Type III ($\rho=0.8$) | (1.81, 2.47, 2.67, 0.33, 2.71), 3 | (2.84, 1.87, 2.65, 0.34, 2.30), 3 | (2.40, 2.88, 2.64, 0.40, 1.67), 3 | (2.63, 2.72, 1.77, 0.35, 2.54), 3 |
| Gene Type IV ($\rho=1$) | (1,3,5,7,9), 3 | (1,3,5,7,9), 3 | (1,3,5,7,9), 3 | (1,3,5,7,9), 3 |

**Table S3**: **Results of the simulation for different scenarios.**

**Scenario 1**: The numbers within each cell represent the number of times the gene was detected within 500 times simulation when the sample size within each class was 5, 10, and 20, respectively.

|  | MICA | min-MCC |
| --- | --- | --- |
| Gene Type I | 246, 427, 497 | 191, 340, 468 |
| Gene Type II | 197, 385, 490 | 78, 73, 87 |
| Gene Type III | 315, 476, 500 | 65, 90, 98 |
| Gene Type IV | 31, 26, 22 | 38, 24, 27 |

**Scenario 2**: The numbers within each cell represent the number of times the gene was detected within 500 times simulation when the number of studies was 2, 4, and 8, respectively.

|  | MICA | min-MCC |
| --- | --- | --- |
| Gene Type I | 281, 427, 495 | 281, 340, 325 |
| Gene Type II | 297, 385, 482 | 297, 73, 0 |
| Gene Type III | 274, 476, 500 | 274, 90, 80 |
| Gene Type IV | 26, 26, 24 | 26, 24, 25 |

**Scenario 3**: The numbers within each cell represent the number of times the gene was detected within 500 times simulation when the number of classes was 2, 3, and 5, respectively.

|  | MICA | min-MCC |
| --- | --- | --- |
| Gene Type I | 191, 427, 500 | 167, 340, 498 |
| Gene Type II | 140, 385, 500 | 122, 73, 129 |
| Gene Type III | 52, 476, 500 | 17, 90, 77 |
| Gene Type IV | 25, 26, 29 | 31, 24, 25 |

**Scenario 4**: The numbers within each cell represent the number of times the gene was detected within 500 times simulation when the concordance level (the pairwise correlation of the mean of each class) was 0.4, 0.6, 0.8, and 1 respectively.

|  | MICA | min-MCC |
| --- | --- | --- |
| Gene Type I ($\rho=0.4$) | 118 | 64 |
| Gene Type II ($\rho=0.6$) | 161 | 77 |
| Gene Type III ($\rho=0.8$) | 176 | 101 |
| Gene Type IV ($\rho=1$) | 500 | 500 |

**Table S4**: **Simulation for equal vs. unequally weighted settings.**

|  | **Study 1** | **Study 2** | **Study 3** | **Study 4** |
| --- | --- | --- | --- | --- |
|  | (n_11_, n_12_, n_13_) =  (5,10,20) | (n_21_, n_22_, n_23_) =  (20,10,5) | (n_31_, n_32_, n_33_) =  (5,10,20) | (n_41_, n_42_, n_43_) =  (20,10,20) |
|  | (μ_11_, μ_12_, μ_13_), σ_1_ | (μ_21_, μ_22_, μ_23_), σ_2_ | (μ_31_, μ_32_, μ_33_), σ_3_ | (μ_41_, μ_42_, μ_43_), σ_4_ |
| Gene Type I | (1,3,5), 3 | (1,3,5), 3 | (1,3,5), 3 | (1,3,5), 3 |
| Gene Type II | (5,3,1), 3 | (5,3,1), 3 | (5,3,1), 3 | (1,7,1), 3 |
| Gene Type III | (1,3,5), 3 | (1,3,5), 3 | (1,7,1), 3 | (1,7,1), 3 |
| Gene Type IV | (0,0,0), 3 | (0,0,0), 3 | (0,0,0), 3 | (0,0,0), 3 |

|  | MICA with equal weight | MICA with unequal weight |
| --- | --- | --- |
| Gene Type I | 428 | 426 |
| Gene Type II | 363 | 359 |
| Gene Type III | 481 | 481 |
| Gene Type IV | 24 | 22 |

**Table S5**: **IPA canonical pathway analysis on M1 and M4 genes in mouse metabolism data.** Top 15 pathways (sorted by the p-value) are listed.

| **Top 15 pathways identified by M1 genes** | **-log(p-value)** | **Top 15 pathways identified by M4 genes** | **-log(p-value)** |
| --- | --- | --- | --- |
| Arsenate Detoxification I (Glutaredoxin) | 5.19 | Superpathway of Methionine Degradation | 3.25 |
| Tetrapyrrole Biosynthesis II | 5.19 | Inhibition of Matrix Metalloproteases | 3.22 |
| Heme Biosynthesis II | 4.28 | Guanosine Nucleotides Degradation III | 2.87 |
| Oxidative Phosphorylation | 4.24 | Hepatic Fibrosis/Hepatic Stellate Cell Activation | 2.84 |
| Acyl-CoA Hydrolysis | 3.66 | Urate Biosynthesis/Inosine 5'-phosphate Degradation | 2.80 |
| Ascorbate Recycling (Cytosolic) | 3.65 | Adenosine Nucleotides Degradation II | 2.69 |
| Neutrophil Extracellular Trap Signaling Pathway | 3.11 | Pulmonary Fibrosis Idiopathic Signaling Pathway | 2.57 |
| TCA Cycle II (Eukaryotic) | 3.00 | Purine Nucleotides Degradation II (Aerobic) | 2.54 |
| Iron Homeostasis Signaling Pathway | 2.89 | Glioma Invasiveness Signaling | 2.43 |
| Necroptosis Signaling Pathway | 2.62 | Methylthiopropionate Biosynthesis | 2.37 |
| Leucine Degradation I | 2.58 | Sulfite Oxidation IV | 2.37 |
| Microautophagy Signaling Pathway | 2.55 | Epithelial Adherens Junction Signaling | 2.33 |
| Role of MAPK Signaling in Promoting the Pathogenesis of Influenza | 2.49 | BEX2 Signaling Pathway | 2.29 |
| Pyrimidine Ribonucleotides Interconversion | 2.30 | NAD Salvage Pathway II | 2.23 |
| Ferroptosis Signaling Pathway | 2.23 | Superpathway of Cholesterol Biosynthesis | 2.17 |

**Table S6**: **LISA results for top 30 ranked transcription factors.** The ranking is obtained by combining Peak-RP method, H3K27ac, DNase-Seq in silico deletion of TF ChIP-Seq peaks.

| Transcription Factor | p-value | Transcription Factor | p-value | Transcription Factor | p-value |
| --- | --- | --- | --- | --- | --- |
| SMC1A | 3.36E-89 | MYC | 9.77E-29 | SP1 | 9.88E-25 |
| CTCF | 1.36E-75 | TFAP2C | 1.54E-27 | NR2F2 | 1.86E-24 |
| NFIA | 2.81E-72 | FOXA1 | 3.85E-27 | BRD4 | 4.01E-24 |
| DPF1 | 1.79E-69 | HIF1A | 7.02E-27 | KDM5B | 4.55E-24 |
| ZMYM3 | 1.88E-62 | TCF7L1 | 1.22E-26 | WDR5 | 5.16E-24 |
| ESR1 | 5.33E-60 | YY1 | 2.26E-26 | TFAP2A | 5.52E-24 |
| MED1 | 5.83E-56 | THAP11 | 5.00E-26 | ERG | 1.13E-23 |
| T | 6.01E-48 | EGR3 | 2.29E-25 | AR | 1.24E-23 |
| BATF3 | 4.06E-46 | MAX | 3.55E-25 | CNOT3 | 2.64E-23 |
| SP140 | 1.61E-34 | E2F1 | 3.72E-25 | SPI1 | 5.44E-23 |

**Table S7**: **Distribution of the number of cells for each cell type.**

|  | B cell | CD4+ T cell | CD8+ T cell | Macrophage | Tumor |
| --- | --- | --- | --- | --- | --- |
| DCIS | 424 | 229 | 168 | 1126 | 3628 |
| Primary | 1695 | 3191 | 4442 | 4534 | 6564 |
| Metastasis | 3147 | 6912 | 1095 | 46 | 62 |

**Table S8. List of immune-tumor discordant genes in TNBC tumor microenvironment.**

| IFI6 | RPL39 | RPS15A | LMNA | FCGR2B | WNK1 | TRIM38 | FOSL2 | DDX6 |
| --- | --- | --- | --- | --- | --- | --- | --- | --- |
| MARCKSL1 | SSR4 | RPL23 | SRP9 | PPP1CB | ITPR2 | IKZF1 | GLS | PCGF5 |
| RPL5 | RPL10 | KRT19 | TM4SF1 | MOB1A | MRPL57 | BTK | CMTM7 | LEMD3 |
| S100A6 | RPS20 | RPL27 | EGR1 | RPL22L1 | RB1 | FLNA | CMTM6 | ACADS |
| FCER1G | RPL7 | PRKAR1A | DEK | AP2M1 | USP22 | MTSS1 | GLB1 | MAP2K1 |
| RPS7 | MTDH | RPL38 | SH3BGRL | FAM53C | GNA15 | MAML2 | SLC25A38 | TXNDC11 |
| EPCAM | RPL30 | ACTG1 | ENY2 | SERPINB6 | NDUFB7 | TRAF3 | PPP4R2 | SLC38A10 |
| RPL37A | COX6C | GNAS | EIF3H | CD83 | ZNF331 | IL16 | CD47 | RAB8A |
| PTMA | RPL8 | PRDX2 | MRPL17 | DDAH2 | DDT | STAT3 | COMMD8 | PARVB |
| RPL32 | IFITM3 | C19orf53 | PLA2G16 | H2AFV | COPA | ABI3 | OCIAD2 | C21orf91 |
| RPL15 | POLR2L | FXYD3 | RNASEH2C | GPAT4 | MARCH1 | LMNB1 | CHIC2 | UBE2E3 |
| GOLGA4 | SPCS2 | RPS11 | MRPL51 | SDCBP | TPBG | MYLIP | NOA1 | GNL1 |
| TMA7 | FDX1 | APP | ZCRB1 | C8orf59 | EIF3E | ELMO1 | PPP3CA | METTL9 |
| RPL29 | RPS25 | RUNX3 | IGHG3 | YWHAZ | SCIMP | KDM6A | SERINC5 | GPR137B |
| C4orf48 | GAPDH | PTTG1 | PSMB3 | CHRAC1 | MX2 | SLC3A2 | BAK1 | DNAJB11 |
| TBC1D1 | MGP | TUBB | COA3 | EEF1D | TRAF3IP3 | ATM | ARHGEF6 | NSD1 |
| ANXA5 | LDHB | HLA-DQB1 | P4HB | ANP32B | TOP2B | ITGB7 | PDLIM2 | NKAP |
| NPM1 | LYZ | CYBB | ANAPC11 | SEC61B | MAP4 | SLFN12L | R3HCC1 | GAPVD1 |
| RPS18 | TAOK3 | KRT7 | ACTN4 | PPP1R14B | TMF1 | CD300A | ATP6V1H | CAMK1D |
| EEF1A1 | RPLP0 | RGCC | RANBP1 | TAF1D | KLHL24 | MAPRE2 | GLIPR2 | ZMYM2 |
| COX7A2 | SAP18 | COTL1 | CAPZA1 | TMEM123 | RASGEF1B | NCF4 | VPS51 | NDE1 |
| SAT1 | PPIB | RUNX1 | RHOC | GLUD1 | SERPINB9 | RAP1A | SRSF8 | PRKCA |
